# METORY: Development of a Demand-Driven Blockchain-Based Dynamic Consent Platform Tailored for Clinical Trials

**DOI:** 10.3389/fmed.2022.837197

**Published:** 2022-05-13

**Authors:** Ki Young Huh, Sang-un Jeong, Seol Ju Moon, Min-Ji Kim, Wooseok Yang, Myeonggyu Jeong, Ildae Song, Yong-Geun Kwak, SeungHwan Lee, Min-Gul Kim

**Affiliations:** ^1^Department of Clinical Pharmacology and Therapeutics, Seoul National University College of Medicine and Hospital, Seoul, South Korea; ^2^Center for Clinical Pharmacology and Biomedical Research Institute, Jeonbuk National University Hospital, Jeonju, South Korea; ^3^Clinical Trial Center, Seoul National University Hospital, Seoul, South Korea; ^4^Department of Pharmaceutical Science and Technology, Kyungsung University, Busan, South Korea; ^5^Department of Pharmacology, School of Medicine, Jeonbuk National University, Jeonju, South Korea; ^6^Research Institute of Clinical Medicine of Jeonbuk National University, Jeonju, South Korea

**Keywords:** blockchain, clinical trials, dynamic consent, mobile application, platform

## Abstract

The recent advent of the dynamic consent concept intensified the data integrity issue in clinical trials. Incorporating blockchain technology into a dynamic consent platform can be a feasible solution. Due to various clinical trial settings, a demand-driven development strategy is required. We developed a blockchain-based dynamic consent platform named METORY tailored for clinical trials. The platform consisted of three parts: web and mobile application user interface, study management platform, and blockchain platform. Hyperledger Fabric, an enterprise-grade private blockchain framework, was used to integrate blockchain into the study consent platform. We conducted user acceptance tests and applied feedback to the improvement of the platform. Identity and role-based access control was constructed by combining mobile-application-based certificate system and access control functionalities in Hyperledger fabric. Data were encrypted using SHA-256 prior to transmission to blockchain server and TLS protocol was used for in-transit encryption. File-system level encryption was separated implemented within the security measures from Amazon RDS. Users' experience in the clinical trial was acceptable in the ease and usefulness of the platform.

## Introduction

Written informed consent, which is mandated under Good Clinical Practice, should be obtained prior to any study-related procedure in clinical trials (1). The principle also obligates obtaining additional consent when the study protocol is amended (1). In practice, the principle has met several concerns with the advent of digitalization in clinical trials (2). A major concern is that traditional “written” consent cannot ensure proper understanding; therefore, the consent process should be dynamic and interactive (2).

Digitalization in clinical trials was accelerated after the coronavirus-19 pandemic in 2019 (3). In particular, electronic consent (eConsent) in clinical trials was actively implemented as the traditional consent process became unavailable during the pandemic (3, 4). Despite the widespread introduction of eConsent, several major issues have been posed (5, 6). One major concern is whether the study participants adequately understand the information (6). Another issue is security and trust, which requires strict control of access systems (6). The issues were aligned with recent discussions in data integrity, which was emphasized in Good Clinical Practice (7).

Attentions to eConsent in clinical trials are currently intense, coupled with novel clinical designs and decentralized clinical trials (5). As considerable data are generated or managed from electronic sources in this environment, conventional paper-based regulations are not properly working under these settings (5). However, there have been no consistent procedures to replace conventional paper consent with the electronic format (8). In addition, issues in the eConsent platform are associated with the design of the platform, wherein the entire information was conferred *via* electronic media (6, 8, 9).

The issues in implementing eConsent are also closely related to recent dynamic consent concepts in clinical trials. Dynamic consent is characterized by granular decisions from the study participants supported by an interactive digital interface (10). As dynamic consent requires point-by-point decisions from the study participants, it inevitably accompanies a larger number of interactions (11). Accordingly, data integrity and security issues could be intensified in implementing dynamic consent in clinical trials.

One approach to overcome the data integrity issue is the utilization of blockchain technology. Key features of blockchain are immutability and traceability of data, which could bolster data integrity in clinical trials (12). Several attempts have been made to integrate blockchain in dynamic consent (13–15). Most of the attempts were related to dynamic consent models in biobanks (13, 15), wherein the dynamic concept was first introduced in contrast to broad consent (16).

Among the advantages of implementing blockchain in clinical trials, patient privacy is of great importance. Cryptographic algorithms intrinsically provided by blockchains could provide stable measures of patient privacy (17). The prototype blockchain bitcoin used 256-bit Secure Hash Algorithm (SHA-256) replacing IP address (17) and implemented in a consent module (18). Various other encryption algorithms are also attempted, and the inherent anonymization following encryption could be well aligned with clinical trials (19).

To the best of our knowledge, attempts at blockchain-based dynamic consent in clinical trials are relatively rare. The incorporation of blockchain in clinical trials has been mostly focused on data management and in the prototype stage (20, 21). The experience of the platforms in prospective clinical trials is even more limited, although evaluation using retrospective clinical trial data was tried (22).

To address complicated considerations in clinical trials (5), we developed a demand-driven blockchain-based dynamic consent platform tailored for clinical trials named METORY. We designed the platform from pragmatic perspectives, followed by iterative platform enhancement. In addition, we implemented the platform in an actual multicenter clinical trial to evaluate real-world user experience.

## Materials and Methods

### Overall Development Strategy

**Figure 1:**
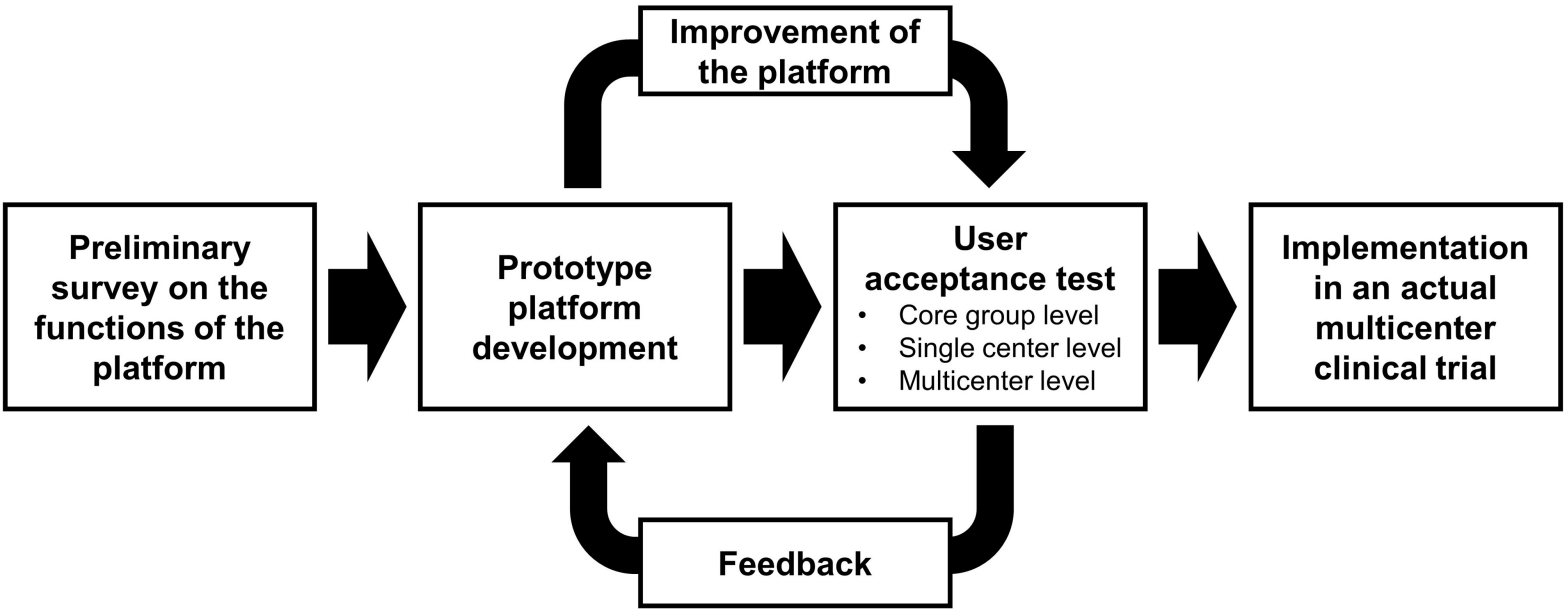
Schematic representation of the development process.

To optimize the development process, we adopted a prototype development approach. Prior to developing a prototype platform, a preliminary survey on the functions of the platform was planned. The prototype platform was built based on the survey results, and three-stage user acceptance tests were performed: core group (mainly with developers), single center, and multicenter levels. The feedback from the user acceptance tests was reflected on the further development of the platform. The final platform was implemented in an actual multicenter clinical trial, and user experience from the trial was collected (Figure 1).

### Preliminary Survey on the Functions of the Platform

To define the main functions of the platform tailored for clinical trials, a preliminary survey was conducted on investigators and personnel in clinical trials. We organized questions with the multimedia components adapted from TransCelerate eConsent guidance (23). The included items were as follows: tiered approach of the interface, video, audio, pictures and diagrams, callout boxes, chats with investigators, knowledge review, section-based participant attestation, and electronic signature. Each question was provided with prototypal illustrations of the system. The questionnaire was sent via group emails of the Korean Society for Clinical Pharmacology, and responses were collected in November 2019.

### Platform Development

**Figure 2:**
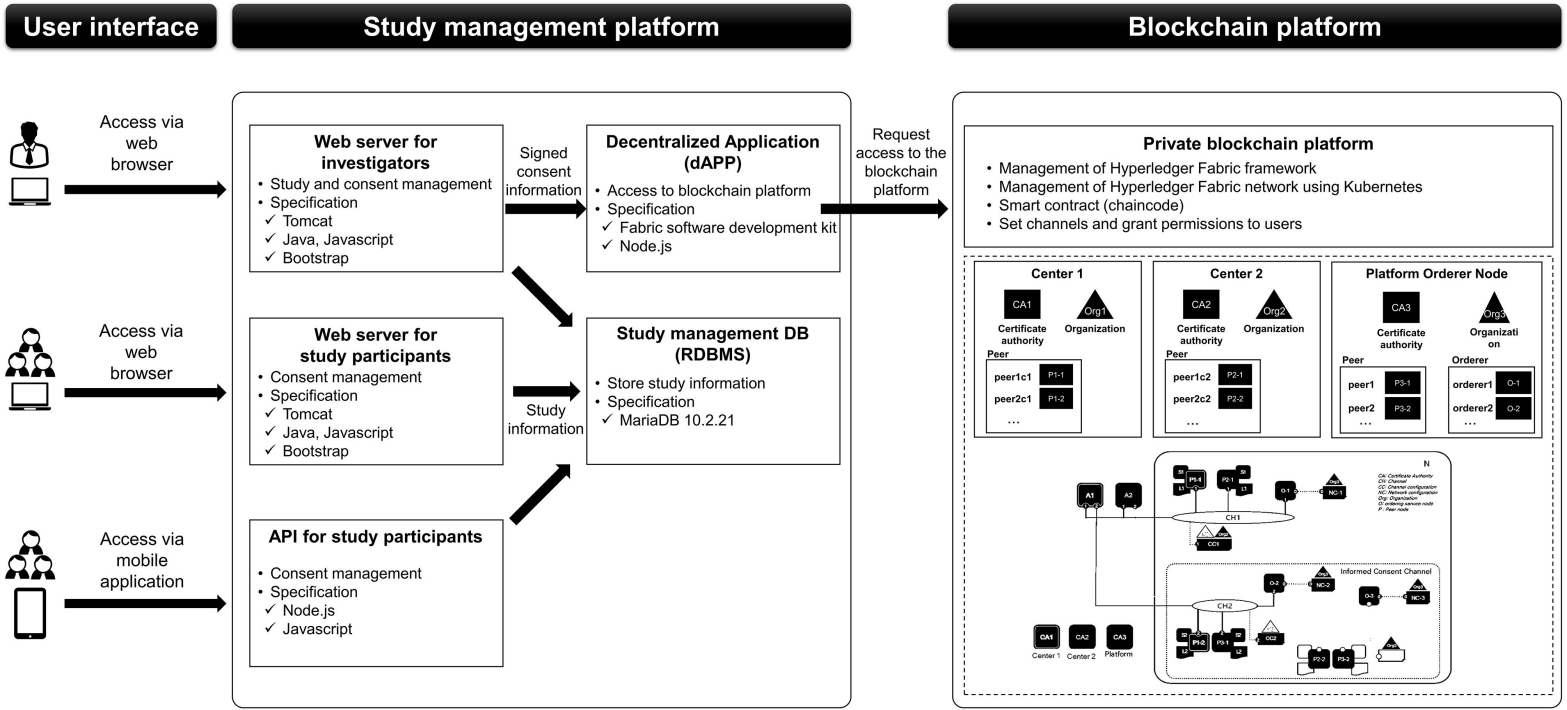
Data flow and architecture of the platform.

The platform was divided into three parts: web and mobile application user interface, study management platform, and blockchain platform. Web and mobile application user interfaces were constructed separately for investigators and study participants. Study management parts comprised the web servers for investigators and study participants, application programming interface (API) for mobile applications, relational database management system (RDBMS) for study management to store study information, and a decentralized application (dApp) server which could access to blockchain platform. Web servers and APIs transmitted *study information* to RDBMS, while the web server for the investigators sent signed consent information to the dApp server. The dApp server requested access to the blockchain platform to record or create transactions or to add signed consent information on the block (Figure 2).

Blockchain platforms could be primarily classified into public, private, or federated blockchain (24). The following characteristics were taken into account on selecting the proper blockchain platform: (i) access control of trusted users, (ii) protocol efficiency, (iii) immutability of data, (iv) management of the platform, and (v) transaction approval rate (24). Availability of smart contract and data security functionalities was also considered as the platform was aimed at dynamic consent.

### User Acceptance Test

User acceptance tests were initially performed with the first prototype platform within the core group consisting of developers and study personnel. The core group evaluated key functions of the platform, such as study, consent, subject management, and electronic consent process. Key functions were described as step-by-step scenarios, and the core group evaluated each step. The results of the user acceptance test were applied to improve the platform. The improved platform was then assessed in study personnel in a single study center, followed by those in another center to evaluate the multicenter scalability of the platform (Table 1).

**Table 1:** User acceptance test scenarios.

**Steps**	**Descriptions**
**Study management by the investigators**	
1	Sign-in with the administrator account
2	Grant an investigators' role
3	Create an account for an investigator
4	Sign-in with the investigator account
5	Modify account information
6	Manage authorization
7	Create an account for a clinical research coordinator
8	Create a study
9	Set up for the study: upload an advertisement for subject recruitment
10	Set up for the study: add descriptions on the advertisement
11	Set up for the study: upload an informed consent form
12	Set up for the study: set the signature format
13	Set up for the study: modify the status of the study
14	Check the advertisement for subject recruitment
**Consent management by the investigators**	
1	Check the subject participation status
2	Check the request for study instruction from the subjects
3	Start instructions on the study
4	End instructions on the stud
5	Sign on the informed consent form
6	Verify access to the blockchain platform
**Subject management by the clinical research coordinators**	
1	Sign-in with the clinical research coordinator account
2	Modify account information
3	Check the subject participation status
4	Check the reservation schedule
5	Answer to the subjects' inquiries
6	Modify the reservation schedule
7	Verify the authentication of a subject
8	Check the request for study instruction from the subjects
9	Check the signed informed consent form
**Consent process by the study participants**	
1	Create a user account
2	Sign-in with the user account
3	Modify account information
4	Check advertisements for subject recruitment
5	Participate in a study
6	Reparticipate in a study
7	Inquire investigators of the study information
8	Make a reservation for a visit
9	Modify the reservation for a visit
10	Authentication
11	Request for instruction from the investigators
12	Get the instruction from the investigators
13	Sign on the informed consent form
14	Review the study record
15	Question and answer using chatting module
16	Review the participation status
17	Sign-out

### User Experience in the Multicenter Clinical Trial

The final platform was implemented in an actual decentralized and multicenter clinical trial using virtual drugs (ClinicalTrials.gov identifier: NCT05047016) (25). The study consisted of 2-week visits and home-based procedures. At the two visits, study participants completed the questionnaires regarding the user experience using the platform. The questionnaire included 5 abbreviated questions at Visit 1 and 16 full questions at Visit 2. Items in the questionnaire were adapted from the mHealth App Usability Questionnaire (26) and modified to be suitable for METORY. The results were summarized descriptively for each item. The clinical trial was approved by the institutional review board of Seoul National University Hospital and Jeonbuk National University Hospital and was conducted in accordance with the Declaration of Helsinki.

## Results

### Preliminary Survey Results

A total of 61 investigators and study personnel responded to the survey. Among the responders, investigators who obtained consent accounted for the largest number (25%), followed by clinical research coordinators (15%) and investigators who did not obtain consent (13%). Most of the participants agreed to the importance of electronic consent (95%).

**Figure 3:**
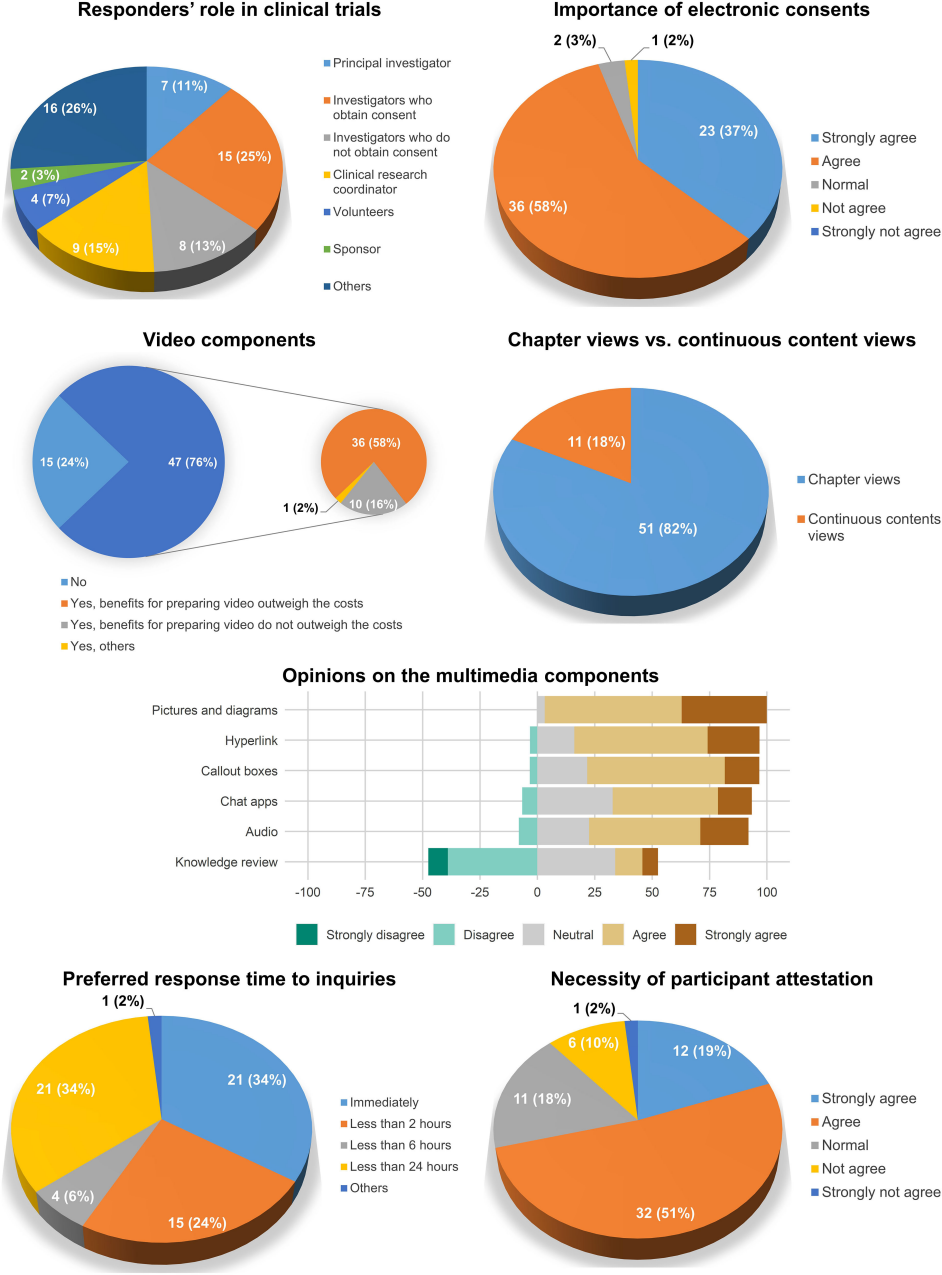
Summary of the preliminary survey on the functions of the platform.

Regarding the contents of the electronic consent system, approximately three-quarters (76%) of the responders agreed to the importance of the video components in electronic consent. The chapter view interface was preferred (82%) to continuous content views (18%). Most of the multimedia components were considered necessary in the electronic consent system except for the knowledge review, wherein negative opinions (47.5%) were greater than positive opinions (18.7%). The opinions on the preferred response time to questions varied: immediately (34%), <2 h (24%), 2–6 h (6%), 6–24 h (34%), others (2%). The necessity of participant attestation (e.g., entering a statement such as “I have no further questions”) was agreed upon in 70% of the responders (Figure 3).

### Study and Consent Management

Study and consent management functions were constructed for investigators. The key functions were the creation and modification of a clinical trial, the management of informed consent forms and information provided to study subjects, and registering the advertisement of subject recruitment. All documents related to clinical trials needed to be approved by the ethical committees and managed in an unmodifiable form (e.g., portable document format).

The principal investigator could grant specific authorizations and functions for the investigators. For example, permissions to provide consent were exclusively granted to the delegated investigators with the physician's license. Other investigators were granted permission to browse the signed consent forms. The status of the consent process was provided as a dashboard to maximize convenience.

**Figure 4:**
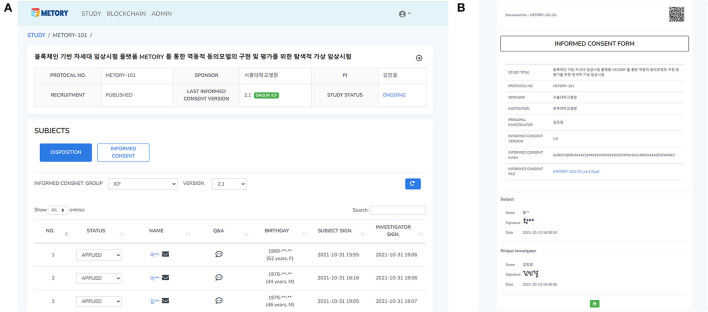
Web interface of the study and consent management: a dashboard for the study management **(A)**, and the signed consent form where hash values were attached **(B)**.

Informed consent forms and information provided to study participants were managed by the version group of the documents. A version group was set separately for each version of the informed consent forms. The original consent form that included the signature from the Institutional Review Board was converted a portable document format file and uploaded. When a study document was uploaded to a platform, the information related to the file (e.g., version and upload date) with the file itself was combined and converted into hash values. As a slight modification in the document could result in considerable changes in hash values, this system could provide the integrity of the data provided to the study participants. The hash values were recorded on the blocks in the blockchain platform. The hash values were stacked in the blockchain platform. The hash values and corresponding QR code were attached to the signed consent form for verification purposes (Figure 4).

### Authentication and Consent Process

**Figure 5:**
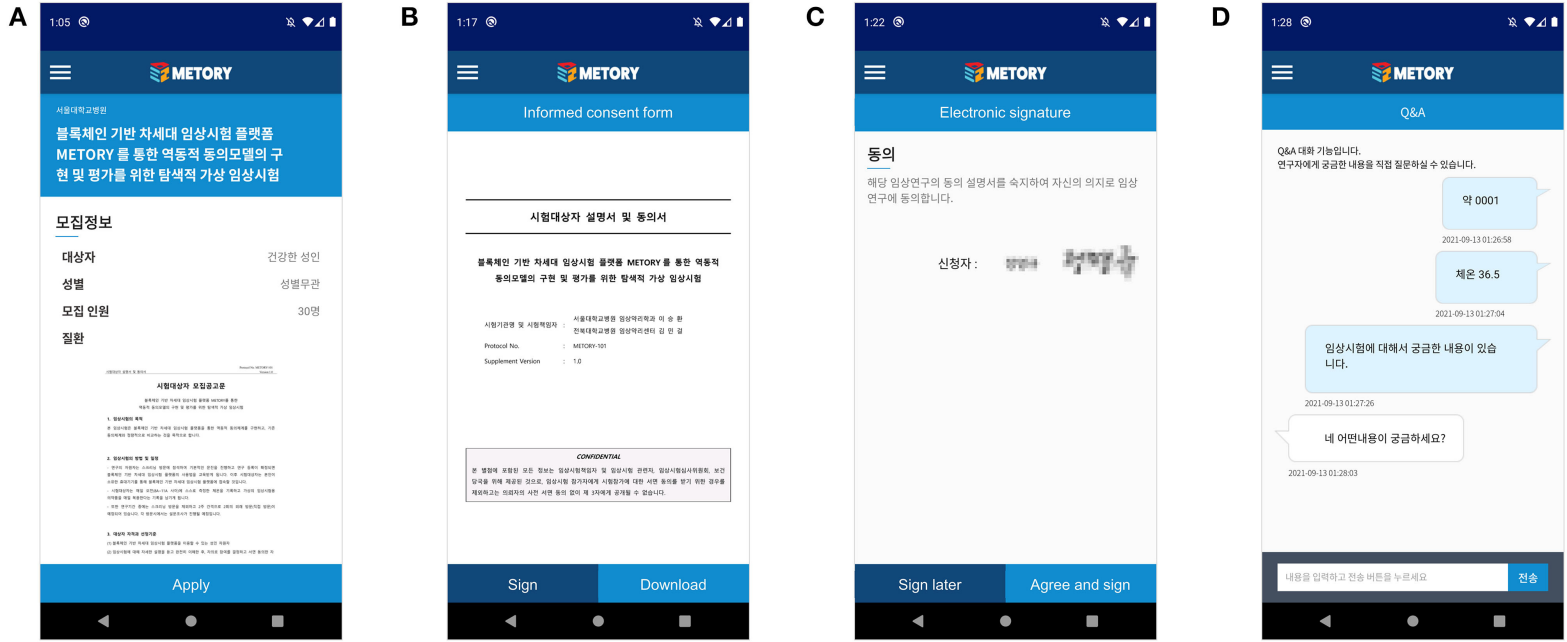
Mobile application interface of the consent process: participation in a study **(A)**, access to the informed consent form **(B)**, electronic signature **(C)**, and chat-ting module between the subject and the investigator **(D)**.

A validated mobile-application-based certificate system broadly used in Korea was utilized for the initial authentication process. After the authentication process was completed, a unique code for each study participant was created and used to identify the study participant. The study participant could select the study to participate in after browsing the advertisement of subject recruitment provided by the investigators. A consent process was initiated after the subject's manifestation of readiness. The investigator then started giving instructions on the study, and the time was recorded. The study participant could read the informed consent form and related study information via the application. In addition, the approved informed consent form could be saved in the study participant's local device. After the instruction was completed, the study participant electronically signed an informed consent form via the application. The investigator could sign an informed consent form that the study participant had signed. Each step of the consent process was recorded on the blockchain platform. The study participant and the investigator could also interact on the chatting system constructed on the application during and after the consent process (Figure 5).

When an informed consent form was amended, investigators could create a new version group of the study. The disclosed study information was then substituted for the information in the latest version group. When the new version group was created, notifications of the new version were sent to the study participants via the application. Each study participant received a push alarm from the application and could access the amended informed consent form. Investigators could also send a text message to the study participants. Each informed consent form was verified by the hash values recorded on the blockchain platform. The study participants provided consent by signing a new informed consent form. The investigators could check the signed status of the version and then sign on the informed consent form, which was transmitted to the blockchain platform to ensure traceability.

### Selection and Integration of Consent Information Into Blockchain Platform

Hyperledger Fabric, an enterprise-grade private blockchain framework (27), was finally selected. We gave the highest importance on the access control, which was recommended in following Good Clinical Practice (7). Access control functionality could not be easily developed using public blockchain platform (e.g., Bitcoin). Given that only trusted subjects and study personnel could participate in clinical trials, the exclusivity of private blockchain could be tolerated. Higher protocol efficiency and rapid transaction approval were also the key elements that were preferred to public blockchains (24). However, immutability of data was inevitably compromised in private blockchains, thus necessitating the use of off-chain storage of data in an external database server (28).

The framework could grant authorization exclusively to members who were enrolled through a trusted membership service provider. This private structure could ensure secure data processing among the participating centers of a clinical trial. The framework allocated nodes to each center, where the study information and user accounts were managed. This could guarantee a decentralized network structure among centers. Channels were used to construct functionalities related to study management; for instance, clinical data storage and consent data storage channels were constructed separately. The blockchain platform was connected to the study management platform by dApp. We used JSP and open JDK 8 for constructing blockchain server on Ubuntu version 18.09 and Apache Tomcat 8. The dApp was developed using software developer kit provided by Hyperledger Fabric using Javascript on Ubuntu version 18.09 and node.js version 10.13.0.

**Figure 6:**
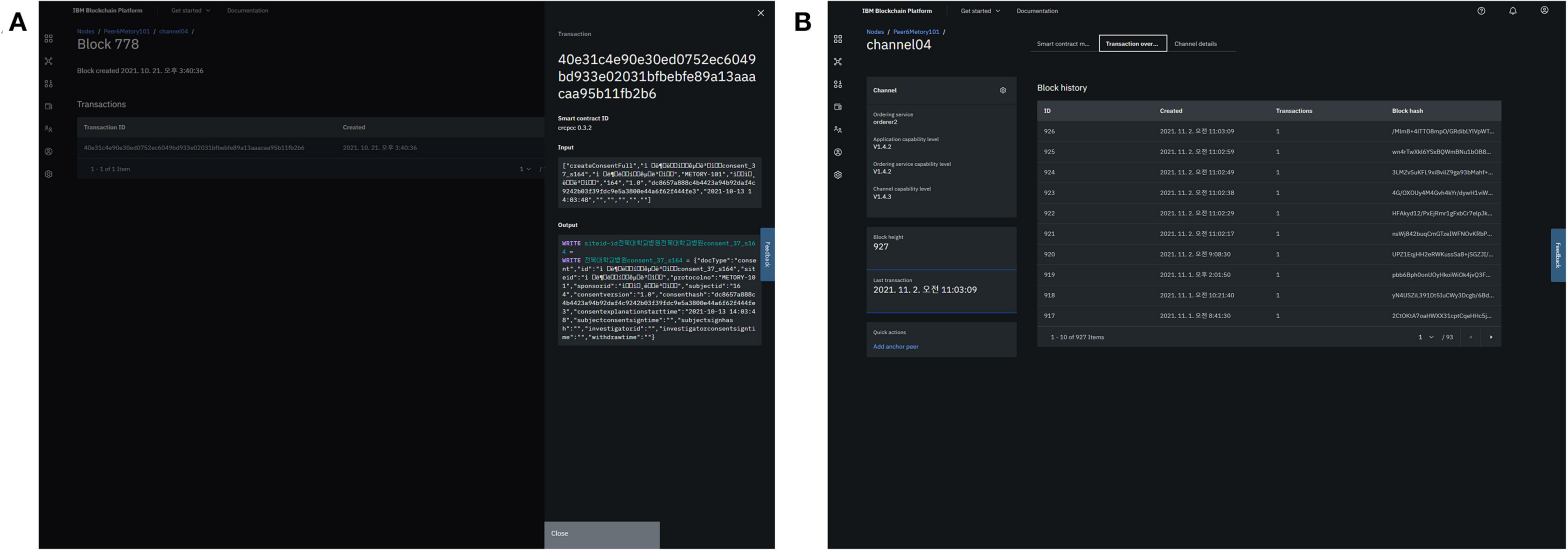
Integration of consent information into the blockchain platform: generation of a transaction **(A)** and appendage of the blocks **(B)**.

The consent data were appended to the blocks as follows. First, the client (a user in the study management platform) converted the consent information (e.g., signature) into a transaction that was compatible with the Hyperledger Fabric system using “chaincode,” a smart contract service provided by the Hyperledger Fabric framework. The transaction was validated by an endorsing peer, and then the results were sent to the client. The client then sent the validated transaction to orderer nodes that distributed the transactions to each peer. Each peer would verify and save the transaction (Figure 6).

Data security was based on a multi-level security approach. Our system allowed insertion of the data only by the approved users. Data created from the study management platform was hashed *via* SHA-256 before calling chaincode. The chaincode also restricted the form of data that could be transmitted to the blockchain platform. Data transmission between peers was encrypted *via* Transport Layer Security (TLS) protocol inside the blockchain platform. As Hyperledger Fabric recommends off-chain data storage in nature (28), file system-level security measures were separately implemented. Database system was constructed using Amazon Relational Database Service (RDS), which provided third-party resource monitoring and snapshot encrypted by Advanced Encryption Standard with a key size of 256 bits (AES-256) (28, 29). In addition, accessible IPs were restricted to authenticated users from the platform by setting security groups.

### User Acceptance Test Results

A total of eight volunteers participated in a core group user acceptance test. The following 4 UAT scenarios were evaluated in the test: study management by the investigators, consent management by the investigators, subject management by the clinical research coordinators, and consent process by the study participants. The feedback was collected as user acceptance test reports and reflected on the platform. The subsequent user acceptance test was conducted on four professional clinical trial personnel in multiple centers. In this test, each tester evaluated both user interfaces for subjects and investigators. The center-level user acceptance test results are summarized in Table 2 by the user interface and functional aspect.

**Table 2:** Summary of the feedbacks from the professionals in the user acceptance test.

**Tester**	**User interface**	**Functions**
1	There was an error when signing in the application due to long input space for personal information. Buttons for functionalities (e.g., reservation for visit, Q&A) were not easily found. Setting dates and times for changing a reservation was not clear.	The modules should deal with multiple versions of informed consent forms because the version of the form could change after protocol amendment. There should be a system to verify whether the subjects read the informed consent form properly and an alarm system to notify participants of changes.
2	Icons and texts might not be visible to the elderly. The alarms for Q&A were not easily seen. The interface of the system was focused on 1:N consent, not for 1:1 consent.	Patients could interact properly with investigators during the verification and recruitment process. There should be restrictions on signatures after the verification of opening and downloading the informed consent form. The term “role” in the application could be confusing.
3	The button for requesting instructions on the study should be more visible. Documents were opened in the current window, which could cause the simultaneous shut-down of the application. Backspace/close/open in a new window buttons should be provided.	A review of the signed consent form should be provided. A review of the previous signed consent form and the modification of the form should be added to the application. Additional consent forms (e.g., consent forms for human-derived materials) should be provided.
4	Setting dates and times for changing a reservation was not clear. Basic information for the functions should be provided. Exit button should be provided.	A review of the signed consent form should be provided. There should a delegation function in the application. A review of the saved document on the blockchain should be provided. Viewing and printing the signed form should be provided.

### User Experience in the Multicenter Trial

A total of 60 subjects were enrolled in the clinical trial (30 subjects in each study center) and participated in the survey. The gender distribution of the subjects was 23:37 (male: female). The mean and standard deviation of the subjects' age was 40.0 ± 10.6 years, while the minimum and maximum were 20 and 67 years, respectively.

In terms of use and satisfaction section, ~90% of the subjects responded within the range of 5–7 (e.g., “The app was easy to use.”). System information arrangement (e.g., “The navigation was consistent when moving between screens.”) and usefulness sections (e.g., “The app improved my access to health care services.”) also showed the similar results. Negative responses were reported to the following items: “I could use the app even when the internet connection was poor or not available” (13.3%) and “This app has all the functions and capabilities I expect it to have (10.0%)” (Table 3).

**Table 3:** Summary of the user experience in the multicenter clinical trial.

**Item**	**Frequency**							
	**1** **Strongly disagree**	**2** **Somewhat disagree**	**3** **Disagree**	**4** **Neutral**	**5** **Agree**	**6** **Somewhat agree**	**7** **Strongly agree**	**Missing**
**Abbreviated questionnaire at Visit 1**	1 (1.7)				4 (6.7)	6 (10.0)	49 (81.7)	
The app was easy to use								
It was easy for me to learn to use the app	1 (1.7)				2 (3.3)	7 (11.7)	50 (83.3)	
The navigation was consistent when moving between screens	1 (1.7)			1 (1.7)	2 (3.3)	10 (16.7)	46 (76.7)	
The interface of the app allowed me to use all the functions offered by the app		2 (3.3)		3 (5.0)	4 (6.7)	13 (21.7)	38 (63.3)	
Whenever I made a mistake using the app, I could recover easily and quickly		1 (1.7)	1 (1.7)	3 (5.0)	6 (10.0)	12 (20.0)	37 (61.7)	
**Full questionnaire at Visit 2**			1 (1.7)		1 (1.7)	9 (15.0)	49 (81.7)	
The app was easy to use								
It was easy for me to learn to use the app			1 (1.7)		2 (3.3)	8 (13.3)	49 (81.7)	
The navigation was consistent when moving between screens				2 (3.3)	1 (1.7)	9 (15.0)	48 (80.0)	
The interface of the app allowed me to use all the functions offered by the app.			1 (1.7)	3 (5.0)	3 (5.0)	15 (25.0)	38 (63.3)	
Whenever I made a mistake using the app, I could recover easily and quickly			1 (1.7)	4 (6.7)	5 (8.3)	9 (15.0)	41 (68.3)	
I like the interface of the app	1 (1.7)		1 (1.7)	4 (6.7)	9 (15.0)	9 (15.0)	36 (60.0)	
The information in the app was well organized, so I could easily find the information I needed			1 (1.7)		8 (13.3)	14 (23.3)	37 (61.7)	
The app adequately acknowledged and provided information to let me know the progress of my action			1 (1.7)	3 (5.0)	4 (6.7)	17 (28.3)	35 (58.3)	
I feel comfortable using this app in social settings				4 (6.7)	7 (11.7)	9 (15.0)	40 (66.7)	
The amount of time involved in using this app has been fitting for me				1 (1.7)	2 (3.3)	8 (13.3)	49 (81.7)	
I would use this app again				6 (10.0)	3 (5.0)	11 (18.3)	40 (66.7)	
Overall, I am satisfied with this app				5 (8.3)	3 (5.0)	12 (20.0)	40 (66.7)	
The app improved my access to health care services				2 (3.3)	10 (16.7)	9 (15.0)	39 (65.0)	
The app helped me manage my health effectively				4 (6.7)	8 (13.3)	13 (21.7)	34 (56.7)	1 (1.7)
This app has all the functions and capabilities I expect it to have			6 (10.0)	4 (6.7)	6 (10.0)	15 (25.0)	28 (46.7)	1 (1.7)
I could use the app even when the internet connection was poor or not available	4 (6.7)	2 (3.3)	2 (3.3)	5 (8.3)	7 (11.7)	13 (21.7)	26 (43.3)	1 (1.7)

## Discussion

We developed a blockchain-based dynamic consent platform tailored for clinical trials. The platform incorporated the private blockchain framework to optimize functions in clinical trials. Based on iterative user acceptance tests, the platform was tuned specifically for clinical trials, including decentralized settings. The user experience for the platform in the real-world implementation was generally positive.

Data security and privacy issues have been of importance in clinical trials using a digital system (30). Angeletti et al. (30) listed three key principles for privacy as follows: (i) privacy of patients and the confidentiality of health care data, (ii) integrity of healthcare data, and (iii) availability of health data for authorized persons. The second and third issue are closely related to authentication and access control issues.

We constructed both identity and role-based access control combining mobile-application-based certificate system and private blockchain framework. The structure was aimed to serve initial identification of participants and restriction of data flow within a clinical trial. We assumed relatively small number of study centers at this stage and preferred private blockchains to consortium ones. Thus, we chose Hyperledger Fabric among private blockchains in that it could easily allow only permissioned users to join after separate identification process. In addition, Hyperledger Fabric has been widely applied for decentralized access control with similar purpose, especially for untrustworthy Internet of Things (IoT) environments (31–33).

Although Hyperldger Fabric has been widely applied for healthcare applications (28, 34), innate architecture-level concerns in the framework should also be considered (35). The security concerns of Hyperledger Fabric are classified into consensus, chaincode, network, and privacy preserving mechanism aspects (35). We paid special attention to the network aspect; Hyperledger Fabric adopts centralized membership service provider which manages registration. When the membership service provider were to be compromised, access control in the whole system would be disrupted (35). This issue is also applied in our platform and needs further investigation.

Another important concern lies in the external access of data through Hyperledger Fabric (36). Hyperledger Fabric supports only text-based data and connection to external database system is frequently recommended (28). In this situation, access to the external files must be consistent among peers in independent environments (36). A recent research found that external database supported in Hyperledger Fabric (e.g., LevelDB and CouchDB) could cause significant overhead, leading to transaction failure (37). As our system also incorporated external database system, the issue also should be solved in further development.

In addition, real-world application of eConsent also required sophisticated consensus. We found that most study personnel agreed to the necessity of eConsent (even prior to the coronavirus-19 pandemic) in our preliminary survey. However, despite the increasing need for eConsent, the format and actual effectiveness of eConsent is still under debate (38, 39). Our survey could give several clues to implementing eConsent in clinical settings, especially for which elements to be incorporated in the eConsent system.

Conveying proper understanding and ensuring security to the study participants are both crucial elements in eConsent. The results of the clinical trial revealed that informed consent that subjects gave did not always mean accurate understanding of the contents (25). The results were contradictory to the subjective feeling of understanding (“*The app adequately acknowledged and provided information to let me know the progress of my action.”*) in the questionnaires. The findings were aligned with the results of a previous randomized clinical trial with 734 patients, where eConsent with trust-enhanced components gave significantly greater satisfaction to patients than standard eConsent (40). Thus, eConsent needs to be implemented in a personalized manner and should balance the amount of information given to not overwhelm study participants (41).

There are several limitations to our studies. The platform we developed did not fully incorporate elements regarded as important in eConsent, such as callout box hyperlinks, due to technical difficulties. In addition, we evaluated the platform only in limited populations. Further investigations in various clinical trials are required. In-depth investigations for data security and privacy need to be performed in the further researches. Nonetheless, we successfully incorporated blockchain into a dynamic consent platform. The user experience in the actual clinical trial was also promising.

In conclusion, we developed a private blockchain-based dynamic consent platform tailored for clinical trials. The platform successfully functioned in an actual multicenter clinical trial with satisfactory user experiences.

## Data Availability Statement

The raw data supporting the conclusions of this article will be made available by the authors, without undue reservation.

## Ethics Statement

The clinical trial was approved by the Institutional Review Board of Seoul National University Hospital and Jeonbuk National University Hospital and was conducted in accordance with the Declaration of Helsinki. The patients/participants provided their written informed consent to participate in this study.

## Author Contributions

KH, S-uJ, Y-GK, SL, and M-GK: conceptualization. KH, SL, and M-GK: methodology. S-uJ, M-JK, and M-GK: software. M-JK, WY, and MJ: validation. KH: formal analysis and data curation. SM and KH: investigation. M-GK and SL: resources and funding acquisition. KH and S-uJ: writing—original draft preparation, visualization, and project administration. IS, SL, and M-GK: writing—review and editing. Y-GK, SL, and M-GK: supervision. All authors contributed to the article and approved the submitted version.

## Funding

This research was supported by a grant from the Korea Health Technology R&D Project through the Korea Health Industry Development Institute (KHIDI), funded by the Ministry of Health and Welfare, and Republic of Korea (Grant Number: HI19C0332).

## Conflict of Interest

The authors declare that the research was conducted in the absence of any commercial or financial relationships that could be construed as a potential conflict of interest.

## Publisher's Note

All claims expressed in this article are solely those of the authors and do not necessarily represent those of their affiliated organizations, or those of the publisher, the editors and the reviewers. Any product that may be evaluated in this article, or claim that may be made by its manufacturer, is not guaranteed or endorsed by the publisher.
